# *Neospora caninum* infection in dairy cattle in Egypt: a serosurvey and associated risk factors

**DOI:** 10.1038/s41598-023-42538-8

**Published:** 2023-09-19

**Authors:** Abdelfattah Selim, Ayed Alshammari, Hattan S. Gattan, Mohamed Marzok, Mohamed Salem, Omar A. AL-Jabr

**Affiliations:** 1 Department of Animal Medicine (Infectious Diseases), Faculty of Veterinary Medicine, Benha University, Toukh, 13736 Egypt; 2 Department of Biology, College of Science, University of Hafr Al-Batin, Hafr Al-Batin, Saudi Arabia; 3 Department of Medical Laboratory Sciences, Faculty of Applied Medical Sciences, King Abdulaziz University, Jeddah, Saudi Arabia; 4 Special Infectious Agents Unit, King Fahad Medical Research Center, King AbdulAziz University, Jeddah, Saudi Arabia; 5 Department of Clinical Sciences, College of Veterinary Medicine, King Faisal University, 31982 Al-Ahsa, Saudi Arabia; 6 Department of Surgery, Faculty of Veterinary Medicine, Kafr El Sheikh University, Kafr El Sheikh, Egypt; 7 Department of Medicine and Infectious Diseases, Faculty of Veterinary Medicine, Cairo University, Cairo, 12613 Egypt; 8 Department of Microbiology, College of Veterinary Medicine, King Faisal University, P.O. Box 400, 31982 Al-Asha, Saudi Arabia

**Keywords:** Parasitology, Risk factors

## Abstract

*Neospora caninum* (*N. caninum*) is one of the causative agents that causing cattle abortion, and severe economic losses. Due to the scarcity of data on *N. caninum* infection in Egyptian cattle, the purpose of this study was to estimate the seroprevalence and determine the risk factors for parasite infection. In four governorates in northern Egypt, 540 blood samples from cattle were taken, and tested using a commercial ELISA kit. The overall seroprevalence of *N. caninum* in examined cattle was 28.89%. A multivariate logistic regression model determined that age (OR = 2.63, *P* < 0.001), manual milking (OR = 1.39, *P* = 0.14), abortion history (OR = 2.78, *P* < 0.0001), repetition of estrus (OR = 2.31, *P* < 0.0001), and contact with dogs (OR = 2.57, *P* < 0.0001) were significant risk factors. The findings proved that *N. caninum* infection was one of the factors contributing to abortion and financial losses in dairy cattle in Egypt. Therefore, the application of sanitary security and control programs is very important in dairy farms.

## Introduction

Neosporosis is a parasitic disease, caused by *Neospora caninum* (Apicomplexa: *Sarcocystidae*), an intracellular protozoan and distributed worldwide^1^. *N. caninum* characterized by Heteroxenous biological cycles. The sexual phase of the *N. caninum* cycle only occurs in dogs, coyotes, Dingoes and grey wolves (*Canis lupus*), and is characterized by the release of oocysts in the faeces while bovines are the primary intermediate hosts of the parasite^2,3^. Transmission of *N. caninum* to cattle can be occur either horizontally by the oocyts ingestion or vertically through the placenta^4^. Because the pathogenesis of neosporosis in cattle is complicated, it is unclear why some animals abort while others do not^5^.

The infected cattle could be suffer from abortion, early foetal losses, neonatal deaths, stillbirths, and embryo reabsorption^5,6^. The dairy industry may suffer financial losses as a result of the infrequent, endemic, or epidemic abortion caused by *N. caninum* that frequently occur during the second trimester of pregnancy^7^. Moreover, there are no definite indicators of protective immunity in recovered animals, and the parasite may persist lifelong in the infected host^5^. Furthermore, there are currently no viable vaccines or treatment for *Neospora* infection^8^. In order to develop and apply strategies to control bovine neosporosis, it is crucial to understand risk factors related to *N. caninum* infection^1^.

In order to identify *N. caninum* infection in animals, a variety of laboratory techniques, including serology, histopathology and molecular techniques are now accessible^9^. Serologic tests, such as the indirect fluorescent antibody test (IFAT), direct agglutination test (DAT), enzyme-linked immunosorbent assay (ELISA), and immunoblotting (IB), were suggested as a diagnostic tool to investigate *N. caninum* antibodies^9–11^. ELISA is the most reliable serologic tool for determining specific antibody titers to *N. caninum*. Additionally, because of its relative quickness, it is more suitable for epidemiologic research^1,12–23^. There are some commercial ELISA assays available for identifying specific antibodies to *N. caninum* in bulk and bovine milk. Hence, bulk milk testing can be used to evaluate levels of *N. caninum* antibody in dairy herds^24^.

Studies have shown that frequent exposure to the sources of infection tends to increase the probability of animals to be seropositive for *N. caninum*^25,26^. The seroprevalence of *N. caninum* varies from 0 to 100% in various of animal species around the world. Egypt has reported a range of seroepidemiological data, including 4.3–8.6% in sheep^27,28^, 4.3% in goats^28^, 38.04% in cattle^29^ and 10.9% in camels^14^. However, there was little comprehensive information on the risk factors related to dairy cattle neosporosis in Egypt prior to our investigation.

Therefore, this study was conducted to assess the existence of antibodies against *N. caninum* and related risk factors in dairy cattle in some Egyptian governorates.

## Materials and methods

### Ethical statement

The Benha University ethical committee for animal studies approved all methods including the handling and collection of blood samples. The cattle owners informally consented for the collection of samples. The Faculty of Veterinary Medicine's ethical committee ensured that all procedures followed the rules and regulations. The ARRIVE criteria were adhered to throughout the research process.

### Study area

**Figure 1:**
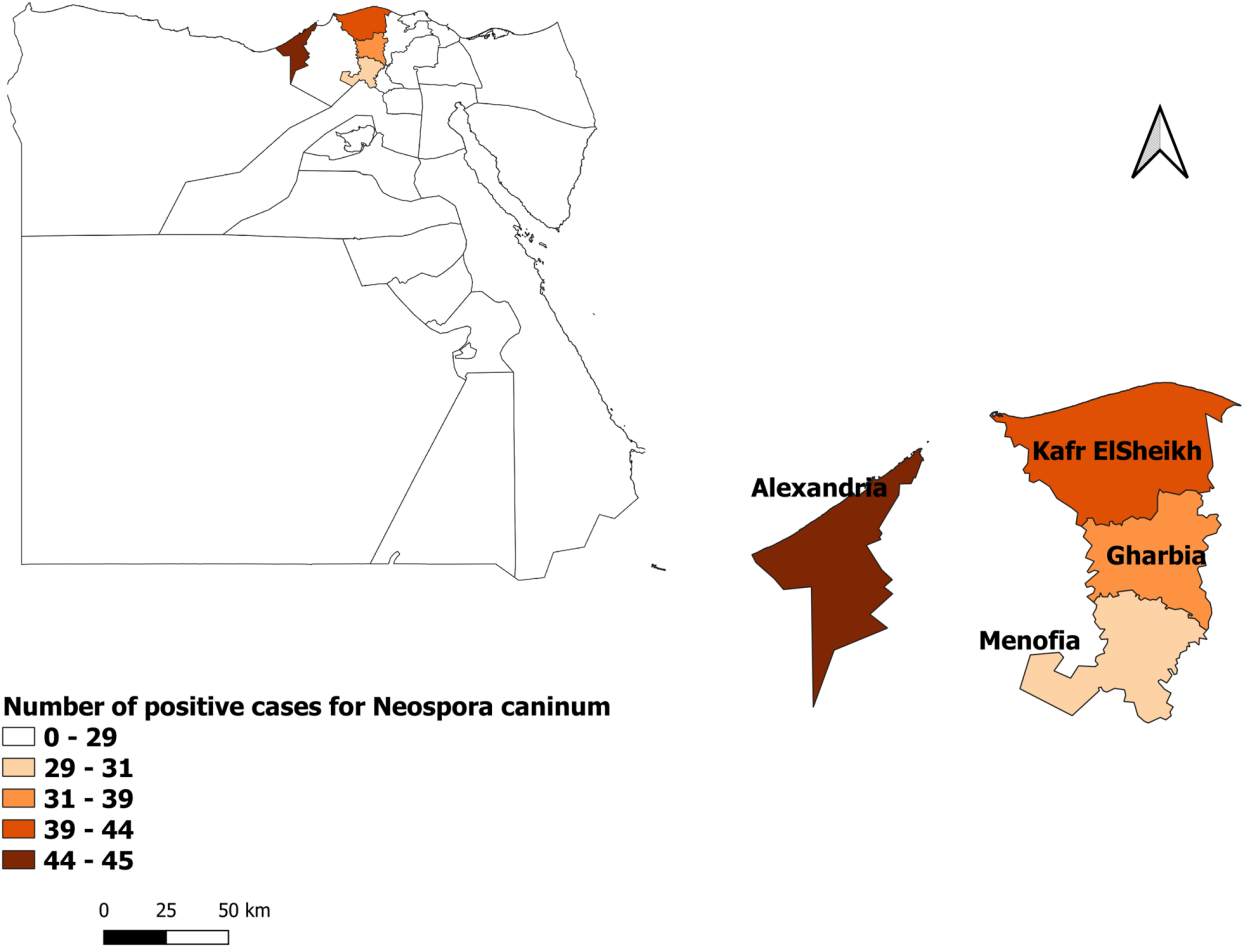
MAP showed the studied areas and prevalence of *N. caninum* (QGIS 3.18.3 software used to generate the MAP).

A cross-sectional investigation was carried out to determine the relation between serological status of *N. caninum* in dairy cattle and possible risk factors. The study was performed in the governorates of Kafr ElSheikh, Menofia, Gharbia, and Alexandria, situated at northern Egypt at 31°06′42″ N 30°56′45″ E, 30.52° N 30.99° E, 30.867° N 31.028° E and 31°11′51″ N 29°53′33″ E, Fig. 1.

The climate of these areas are usually warm with an average annual temperature of 25 °C, relative humidity between 40 and 60% with moderate rainfall 100-mm per year. Because of this, the study region is suitable for farming and raising animals, especially dairy cows.

### Animals and sampling

The study was performed during 2021 to determine the presence of anti- *N. caninum* antibodies among cattle raising in four Egyptian governorates. The sample size needed to determine seroprevalence was estimated according to the Thrusfield formula^30^, based on previous prevalence rate in cattle 38.04% reported by Gaber et al.^29^, precision 10% and level of confidence 95%. A total of 540 blood samples were randomly collected from dairy cattle using simple random sampling. For each examined cattle, the veterinarian and owner filled out a brief questionnaire that was used to gather data at the time of sampling. According to collected data, animals were classified based on location (Kafr ElSheikh, Gharbia, Menofia and Alexandria), age (< 2, 2–4 and > 4 years old), sex (female and male), breeding service (natural or artificial insemination), milking (automated or manual), stage of pregnancy (1–3, 4–6 and 7–9 months), parity (primiparus and pluriparus), gestation (yes or no), abortion history (yes or no), repeat of estrus (yes or no), retention of placenta (yes or no) and dog contact (yes or no). Using vacutainer tubes, five mL of blood were drawn from the jugular vein. The blood was then allowed to coagulate before being centrifuged at 3000×*g* for 10 min to separate the serum, then it was stored until use at − 20 °C.

### Serological analysis

All animals’ sera were examined for anti-IgG to *N. caninum* using an ELISA kit (ID Screen Neosporosis indirect multi-species; ID-Vet, France) in accordance with the manufacturer's instructions.. Each sample’s optical density (OD) was determined using microplate reader at 450 nm, and seropositive animals (Sp) were identified using the computation of the S/P%, whereas serum samples were regarded as positive if the S/P% was more than 50%.

### Statistical analysis

The statistical software SPSS version 24 (IBM, SPSS Inc., Chicago, IL, USA) was used for all calculations. The relation of neosporosis with various risk factors was assessed using the non-parametric Chi-square test. Significant results were defined with *P* value < 0.05. All variables that had a *P* < 0.25 in the univariate analysis were subjected to the multivariate logistic regression model to evaluate the independent risk factors of each variable. Using multivariate logistic regression, the odds ratio (OR) and the corresponding 95% CI were determined^19,21,31–34^. The Hosmer–Lemeshow statistic was computed to assess the model's goodness-of-fit^35^.

## Results

Out of 540 examined cattle, 156 had *N. caninum* antibodies, with overall seroprevalence rate of 28.89%. The seroprevalence rate in Kafer ElSheikh was not substantially greater than that in the other locations under study, Table 1.

**Table 1:** Seroprevalence of *Neospora caninum* in cattle raising in the four governorates under the study.

Variable	Total No of examined cattle	No of positive	No of negative	%	95%CI	Statistic
Locality						
Kafr ElSheikh	130	43	87	33.08	25.58–41.55	χ^2^ = 2.291 df = 3 *P* = 0.514
Gharbia	140	38	102	27.14	20.46–35.04	
Menofia	120	30	90	25.00	18.11–33.44	
Alexandria	150	45	105	30.00	23.24–37.76	
Age						
< 2	140	22	118	15.71	10.61–22.64	χ^2^ = 16.238 df = 2 *P* < 0.0001*
2–4	210	68	142	32.38	26.42–38.98	
> 4	190	66	124	34.74	28.33–41.75	
Breeding service						χ^2^ = 1.218 df = 1 *P* = 0.270
Bull	210	55	155	26.19	20.71–32.53	
AI	330	101	229	30.61	25.88–35.78	
Milking						
Automated	180	40	140	22.22	16.76–28.84	χ^2^ = 5.841 df = 1 *P* = 0.016*
Manual	360	116	244	32.22	27.6–37.21	
Parity						
Primiparus	110	29	81	26.36	19.03–35.29	χ^2^ = 0.429 df = 1 *P* = 0.513
Pluriparus	430	127	303	29.53	25.41–34.01	
Gestation						
Yes	350	95	255	27.14	22.75–32.03	χ^2^ = 1.476 df = 1 *P* = 0.224
No	190	61	129	32.11	25.88–39.05	
Stage of gestation (months)						
1–3	75	18	57	24.00	15.75–34.78	χ^2^ = 0.945 df = 2 *P* = 0.623
4–6	180	48	132	26.67	20.75–33.57	
7–9	95	29	66	30.53	22.18–40.4	
History of abortion						
Yes	120	58	62	48.33	39.58–57.18	χ^2^ = 28.395 df = 1 *P* < 0.0001*
No	420	98	322	23.33	19.54–27.61	
Repetition of estrus						
Yes	310	110	200	35.48	30.36–40.95	χ^2^ = 15.409 df = 1 *P* < 0.0001*
No	230	46	184	20.00	15.34–25.64	
Placental retention						
Yes	180	46	134	25.56	19.74–32.4	χ^2^ = 1.460 df = 1 *P* = 0.227
No	360	110	250	30.56	26.03–35.5	
Contact with dogs						
Yes	350	120	230	34.29	29.51–39.41	χ^2^ = 14.103 df = 1 *P* < 0.0001*
No	190	36	154	18.95	14.01–25.12	
Total	540	156	384	28.89	25.23–32.85	

*The result is significant at *P* < 0.05.

The statistical findings regarding the risk variables for seroprevalence showed no significant relationship between breeding service, parity, gestation, retained placenta and stage of gestation and seroprevalence of *N. caninum* (*P* > 0.05), Table 1.

Regarding of cattle age, there was a highly significant (*p* < 0.05) correlation between age and seroprevalence. Young cattle (< 2 years) had a significantly lower seroprevalence (15.71%) than older cattle (32.38%) in cattle of 2–4 years and 34.74% in cattle of > 4 years. For milking, the higher seroprevalence was found in cattle subjected to manual milking (32.22%) when comparing to those subjected to automated milking (22.22%; *P* < 0.05), Table 1.

The prevalence of *N. caninum* was significantly correlated with the history of abortion in females. The prevalence in this group was higher (48.33%) than in cows without a history of abortions (23.33%; *P* < 0.0001). Of the 310 females with a history of recurrent estrus, 110 (35.48%; *P* < 0.0001) were seropositive for *N. caninum*, whereas only 46 (20%) of the 230 females without a history of repeated estrus were seropositive, Table 1.

Considering contact of examined cattle with dogs, strong significant association was found between cattle contact with and seroprevalence, the highest seroprevalence rate was reported in cattle contact with dogs (34.29%) when comparted to other animals, Table 1.

The multivariable logistic regression model was applied to the variables in the univariable analysis that had a *P* < 0.25. The findings showed that the odds of contracting *N. caninum* infection were three times higher in adult cattle older than four years (OR = 2.63, 95% CI: 1.47–4.71), one time higher in manual milking (OR = 1.39, 95% CI: 0.89–2.17), three times higher in cattle with a history of abortion (OR = 2.78, 95% CI: 1.76–4.41), two times higher in cattle suffering from repetition of estrus (OR = 2.31, 95% CI: 1.52–3.53) and three times higher in cattle contact with dogs (OR = 2.57, 95% CI: 1.63–4.05), Table 2.

**Table 2:** Multivariable logistic regression analysis for risk factors associated with *Neospora caninum* infection.

Variable	B	S.E	OR	95% CI for OR		*P* value
				Lower	Upper	
Age						0.005
2–4	0.717	0.292	2.05	1.16	3.63	0.014
> 4	0.969	0.297	2.63	1.47	4.71	0.001
Milking						
Manual	0.331	0.228	1.39	0.89	2.17	0.146
History of abortion						
Yes	1.023	0.235	2.78	1.76	4.41	< 0.0001
Repetition of estrus						
Yes	0.838	0.216	2.31	1.52	3.53	< 0.0001
Contact with dogs						
Yes	0.944	0.232	2.57	1.63	4.05	< 0.0001

B: Logistic regression coefficient, SE: standard error, OR: odds ratio, CI: confidence interval.

## Discussion

*Neospora caninum* is one important cause in cattle abortion^36^. Analyzing seroprevalence, and consequently the exposure of dairy cattle populations to *N. caninum*, is crucial for determining populations that may be susceptible to neosporosis and for looking into the probable modes of the parasite's transmission. The prevalence and risk factors of cow neosporosis must be understood in order to develop and implement control programme measures^26,37,38^.

In Egypt, the antibodies against *N. caninum* were detected in some species like sheep, cattle and camels^14,27,29^ but the epidemiological data about the disease in cattle is very limited and restricted in some areas in Egypt. Thus, this study aimed to investigate the seroprevalence of *N. caninum* in dairy cattle and assess the potential risk factors associated to infection.

In the present study, there were 28.89% of animals that tested positive for *N. caninum,* which come in agreement with prevalence rate 28.3% reported in Colombia^39^.

Neosporosis in globally distributed and the reported prevalence rates range from 10.7 to 19.6% in Africa^40,41^, 5.7–43% in Asia^42,43^, 7.6–76.9% in America^44,45^ and 0.5–27.9% in Europe^46,47^. However, the reported rate in the present study is not high, similar findings were reported in Brazil^48^ and Sengal^49^.

Among the study's governorates, the seroprevalence rate of *N. caninum* varied non-significantly and Kafr ElSheikh had the highest rate in comparison with other areas. These results concurred with those of Gaber et al.^29^, they reported that Kafr ElSheikh had a high incidence of *N. caninum*. This could be explained by the fact that this governorate’s management, climate and environmental factors play a significant effect in the survivability of *N. caninum* oocysts^33,50–60^.

Neosporosis in globally distributed and the reported prevalence rates range from 10.7 to 19.6% in Africa^40,41^, 5.7–43% in Asia^42,43^, 7.6–76.9% in America^44,45^ and 0.5–27.9% in Europe^46,47^. However, the reported rate in the present study is not high, similar findings were reported in Brazil^48^ and Sengal^49^.

These variances could be brought about by alterations in the climate, study design, detection techniques, farm management, sample size, and varying degrees of exposure to risk factors^17,18,23,59,61,62^.

Studies have shown that frequent exposure to the sources of infection tends to increase the probability of animals to be seropositive for *N. caninum*^25,26^. According to Moore et al.^63^, the risk of seropositivity increased 3.5% for every year that bovine and buffalo ages increased. Our findings are in line with earlier research and demonstrate that elder cattle > 4 years were more likely than younger to have infection with sporulated oocysts of *N. caninum*. Contrarily, other studies from various countries, including Brazil^64,65^, Croatia^66^, Jordan^67^, Romania^46^, and Venezuela^68^, found no correlation between age and *N. caninum* infection, indicating that transplacental transmission is likely more significant for these herds. Our findings suggest that horizontal transmission is also a significant factor in the epidemiology of *N. caninum* in cattle, despite the fact that vertical transmission is typically prove to be the main route of transmission in cattle^15,69,70^.

In cow neosporosis, the semen plays a significant role in disease transmission. Compared with cattle bred naturally from Iran (17.1%) and Spain (7.4%)^71,72^, pregnant heifers undergoing artificial insemination (AI) had higher levels of IgG against *N*. *caninum*. The artificial insemination of seropositive dairy cows with beef bull semen may affect the role of the placenta as a result of crossbreeding^73^. Okumu et al.^74^ found that abortion was considerably higher in pregnant cows with AI when there was no quick testing for cow neosporosis on the semen donors.

Interestingly, cattle were subjected to manual milking showed significant higher seroprevalence than those subjected to automated milking, which come in accordance with findings of Llano et al.^39^. This attributed to poor hygienic condition and contamination of milker’s hand by feces contain sporulated oocyst have significant role in horizontal infection transmission during milking^75^.

It is generally established that seropositive *N. caninum* cattle are more likely to prone abortion than seronegative *N. caninum* cattle^1^. We found that the proportion of seropositive cows that had a history of abortion (48.33%) was substantially higher than the proportion of seronegative cows (23.33%) in a group of cows with the same clinical symptoms. This gives circumstantial evidence that *N. caninum* may contribute to cow abortions in the area under study. These fundamental conclusions concur with those made by Llano et al.^39^ in Colombia.

Furthermore, a considerable percentage of recurrent estrus cattle (35.48%;* P* < 0.0001) had anti-*N. caninum* antibodies. Similar results were found in a study carried out in the southeast of Brazil, where animals with repeat oestrus and transient anoestrus were 3.8 and 3.4 times, respectively, greater likelihood of seropositivity than those without the same clinical indications^76^.

In the present study, cattle suffered from repeat breading and early embryonic death had high seropositivity for *N. caninum* infection which come in agreement with prior findings of Buxton et al.^77^ and^78^. This could be as a result of the fetus’s immature immune system and lesions induced by parasites in the placental tissues, which result in early embryonic mortality and the return to oestrus^77^. This theory is consistent with research from Australia and Senegal that found that seropositive animals for *N. caninum* needed more inseminations to conceive, which is related to embryonic loss in the early stage of pregnancy^49,79^.

Similar to the findings of Barling et al.^80^, *N. caninum* infection in dairy cattle had a substantial correlation with close contact with dogs. This might be due to eating of aborted materials by dogs, which play an important role in horizontal transmission of infection to susceptible animals^81,82^. In Canada, Vanleeuwen et al.^83^ verified that there is a higher risk of infection on properties with dogs who have access to placentas and foetuses than on properties where dogs are not permitted to come into contact with these materials. In this area, preventive actions are advised to reduce the likelihood of dogs consuming contaminated bovine tissues. Since the dogs on one property frequently visited the neighbors’ properties, the close proximity of the farms also made it impossible to get reliable information about the canine population^39^.

## Conclusion

*N. caninum* seroprevalence and distribution throughout all examined areas confirm that the parasite is common in Northern Egypt. Concerning to risk factors associated with *N. caninum* infection, the higher seroprevalence was observed in elder cattle, subjected to manual milking, with history of abortion or repetition of estrous and close contact with dogs. Further studies are necessary to examine sanitary application in dairy farms and to implement an efficient control program.

## Data Availability

This article contains all of the data that was created or analyzed throughout the investigation.
